# Integrating human omics data to prioritize candidate genes

**DOI:** 10.1186/1755-8794-6-57

**Published:** 2013-12-18

**Authors:** Yong Chen, Xuebing Wu, Rui Jiang

**Affiliations:** 1 Department of Automation, MOE Key Laboratory of Bioinformatics; Bioinformatics Division and Center for Synthetic & Systems Biology, TNLIST, Tsinghua University, Beijing 100084, China; 2 Institute of Biophysics, Chinese Academy of Sciences, Beijing 100101, China; 3 David H. Koch Institute for Integrative Cancer Research, Massachusetts Institute of Technology, Cambridge, MA 02139, USA; 4 Computational and Systems Biology Graduate Program, Massachusetts Institute of Technology, Cambridge, MA 02139, USA

## Abstract

**Background:**

The identification of genes involved in human complex diseases remains a great challenge in computational systems biology. Although methods have been developed to use disease phenotypic similarities with a protein-protein interaction network for the prioritization of candidate genes, other valuable omics data sources have been largely overlooked in these methods.

**Methods:**

With this understanding, we proposed a method called BRIDGE to prioritize candidate genes by integrating disease phenotypic similarities with such omics data as protein-protein interactions, gene sequence similarities, gene expression patterns, gene ontology annotations, and gene pathway memberships. BRIDGE utilizes a multiple regression model with lasso penalty to automatically weight different data sources and is capable of discovering genes associated with diseases whose genetic bases are completely unknown.

**Results:**

We conducted large-scale cross-validation experiments and demonstrated that more than 60% known disease genes can be ranked top one by BRIDGE in simulated linkage intervals, suggesting the superior performance of this method. We further performed two comprehensive case studies by applying BRIDGE to predict novel genes and transcriptional networks involved in obesity and type II diabetes.

**Conclusion:**

The proposed method provides an effective and scalable way for integrating multi omics data to infer disease genes. Further applications of BRIDGE will be benefit to providing novel disease genes and underlying mechanisms of human diseases.

## Background

The identification of disease-associated genes is the primary step towards the explanation of pathogenesis of human complex diseases. Functional genomics have enabled the use of large-scale molecular and physiological data for not only the identification of causative genes associated with a disease but also the discovery of gene modules that directly respond to genetic and environmental perturbations associated with the disease [1-3]. For example, Freudenberg and Propping proposed to prioritize candidate genes according to gene semantic similarities derived from the gene ontology [4]. Perez-Iratxeta *et al*. utilized the fuzzy set theory to construct a scoring system for discovering disease-related genes based on literature descriptions of diseases and functional annotations of genes [5]. Methods have also been proposed to make use of such high-throughput data as protein-protein interactions and gene expression profiles [6-10]. Moreover, methods have also been proposed to integrate multiple data sources for the purpose of achieving highly accurate identification of genes involved in diseases or biological processes [11]. A common characteristic of these methods is the requirement of a set of genes known as associated with a query disease before the inference of novel associations between the query disease and candidate genes. Nevertheless, according to the recent release of the OMIM (Online Mendelian Inheritance in Man) database [12], genetic bases for a significant proportion of known diseases are completely unknown, and thus applications of these methods are greatly restricted.

To overcome this limitation, methods have been proposed to utilize disease phenotypic similarities data with protein-protein interaction (PPI) data for the prioritization of candidate genes [13,14]. It has been shown that human inherited diseases may overlap in their clinical traits, described in databases such as OMIM. Moreover, based on the overlapping of clinical traits, phenotypic similarity between diseases can be derived [15,16] and used with the proximity of gene products in a PPI network to discover disease genes. For example, Lage *et al*. proposed a Bayesian model to integrate phenotypic similarities and PPI data [14]. Wu *et al*. developed a method called CIPHER to explain phenotypic similarities using gene proximities [13]. Wu *et al*. also proposed a method named AlignPI to align a phenotype network against a PPI network [10]. Li and Patra adopted a random walk model named RWRH to derive strength of associations for candidate genes with diseases [17]. Vanunu *et al*. put forward a method called PRINCE to simulate how disease status propagated through candidate genes [18]. Chen *et al*. proposed a maximum flow model called MAXIF to calculate strength of associations between a query disease and a set of candidate genes [19]. It has also been shown in these studies that genes associated with similar diseases have both a higher likelihood of physical interactions between their products and a higher similarity of their transcript expression profiles [1,3]. This finding, also referred to as the modular nature of human genetic diseases, has been supported by a number of reports [20-22], suggesting that causative genes for phenotypically similar diseases may reside in the same biological module, either a pathway [23], protein complex [14,24] or a subnetwork of protein interactions [2,25]. Indeed, genes involved in similar diseases also share similar annotations in the gene ontology (GO) [26] and membership in KEGG pathways [27], implying a positive correlation between gene–gene relatedness and disease–disease similarity [28-30].

Motivated by the above understanding, we built in this paper a multiple regression model named BRIDGE (Based on Regression to Identify Disease GEnes) that explains disease similarities by combining functional similarity information of genes derived from such data sources as protein-protein interactions, gene sequence similarities, gene expression patterns, gene semantic similarities, and gene pathway membership relatedness. Serving as an effective information fusion method, our method automatically inferred the relative contribution of each data source and calculated strength of association between a given query disease and a candidate gene. We performed large-scale validation experiments and showed that BRIDGE can rank disease genes at top 1 in 892 out of 1,428 linkage intervals (62.47%). We also showed the capability of our method in prioritizing candidate genes for diseases whose genetic bases are completely unknown. We further performed two case studies on obesity and diabetes to demonstrate applications of our method to complex diseases. We also provided a user-friendly web interface of BRIDGE at http://bioinfo.au.tsinghua.edu.cn/bridge.

## Methods

### Overview of BRIDGE

**Figure 1:**
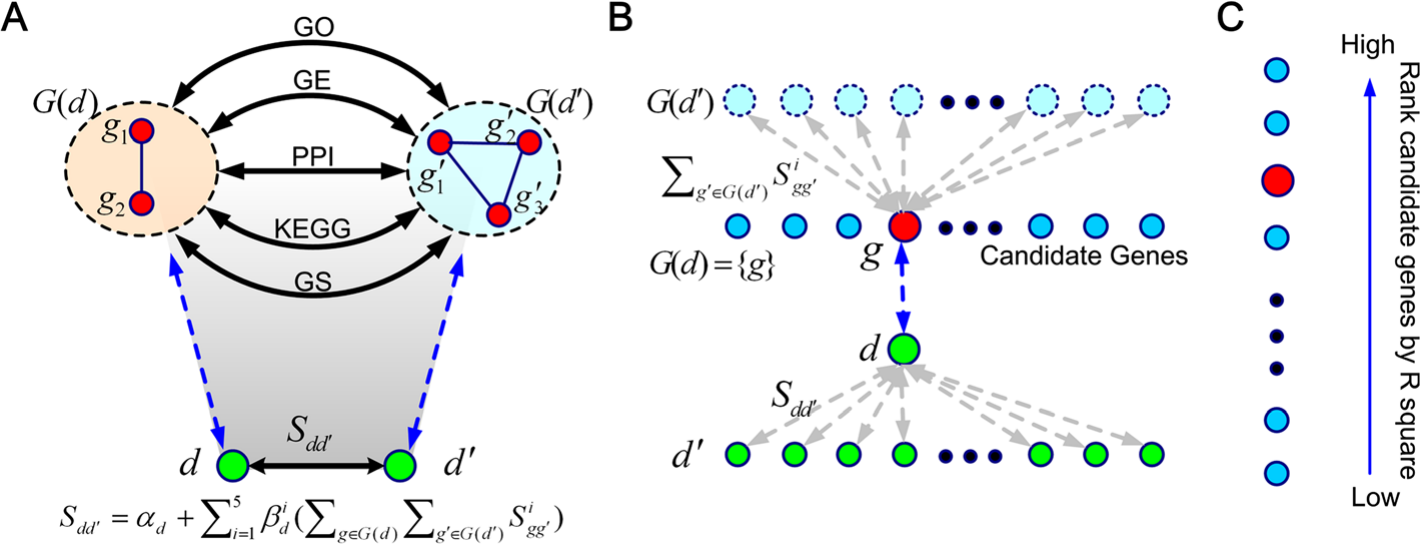
**Scheme of BRIDGE. (A)** A multiple linear regression model is proposed to explain the phenotypic similarity between two diseases using functional similarities between the two sets of genes associated with the diseases. The regression function is $S_{dd^{\prime }}=\alpha_{d}+\sum_{i=1}^{5}\beta_{d}^{i}\left(\sum_{g\in G\left(d\right)}\sum_{g^{\prime }\in G\left(d^{\prime }\right)}S_{gg^{\prime }}^{i}\right)$, where *S*^*i*^_*dd’*_ is the phenotypic similarity between two diseases *d* and *d’*, *S*^*i*^_*gg’*_ the functional similarity between two genes *g* and *g’* derived from the *i*-th data source, *G*(*d*) and *G*(*d’*) genes associated with diseases *d* and *d’*, respectively. We consider five genomic data sources (PPI, GS, GE, KEGG, and GO) in our model. **(B)** Given a query disease *g* and a candidate gene *d*, we assume the candidate gene is the only one associated with the disease, i.e. *G*(*d*) = {*g*}, and we calculate the coefficient of determination (R^2^) of the fitted model as a score to measure the strength of association between the disease and the gene. **(C)** Repeating **(B)** for every candidate gene, we obtain a score for each candidate. We then rank the candidate genes in non-increasing order according to their scores to obtain a ranking list.

The proposed method is based on the assumption that genes involved in diseases with similar phenotypes often share similar characteristics across multiple genomic data sources. Therefore, the phenotypic similarity between two diseases that calculated from text mining can be explained using functional similarities of genes involved in the diseases. The principle of our method is shown in Figure 1(A). Given a disease *d*, we identify a set of genes associated with this disease as *G*(*d*). For another disease *d’*, we identify genes associated with this disease as *G*(*d’*). We then extract the phenotypic similarity score *S*_*dd’*_ for any two diseases that was calculated by using text mining method [31]. Based on a genomic data source indexed by *i*, we calculate a score *S*^*i*^_*gg’*_ to characterize the functional similarity between a pair of two genes g∈*G*(*d*) and g’∈*G*(*d’*), and we compute the summation ∑g∈Gd∑g'∈Gd'Sgg'i to characterize the functional similarity between the two sets of genes *G*(*d*) and *G*(*d’*). In our method, we consider five genomic data sources, including protein-protein interactions (PPI), gene sequences (GS), gene expression profiles (GE), pathway annotations (KEGG), and gene ontology annotations (GO). With these scores calculated, we adopt a multiple linear regression model to explain the phenotypic similarity between the two diseases *d* and *d’* using the functional similarity between the genes involved in these diseases, as Sdd'=αd+∑i=15βdi∑g∈Gd∑g'∈Gd'Sgg'i_,_*α*_*d*_ is the regression intercept and $\beta_{d}^{i}$ (*i* = 1,…,5) the regression coefficients.

Based on this principle, we illustrate in Figure 1(B) the method for calculating a score for an individual candidate gene. Given a query disease *d* and a candidate gene *g*, we assume the candidate gene is the only one associated with the disease, i.e. *G*(*d*) = {*g*}_._ We rewrite the regression function as Sdd'=αd+∑i=15βdi∑g'∈Gd'Sgg'i_,_ where *d’* is any disease included in the phenotypic similarities. We then fit this model using the lasso penalty strategy to automatically filter out unimportant data sources and further calculate the coefficient of determination (*R*^2^) as a score to measure the strength of association between disease *d* and gene *g*. Finally, as illustrated in Figure 1(C), repeating the above procedure for every candidate gene, we obtain a score for each candidate gene. We then rank the candidate genes in non-increasing order according to their scores to obtain a ranking list. The details of calculations of gene similarities in each datasets, disease similarities and linear regression with lasso penalty are explained in the following subsections.

### Derivation of gene similarity and disease similarity

We use gene functional similarities to quantify the degree of sharing common characteristics between pairs of genes. A gene functional similarity provided for a pair of genes is a score that ranges from 0 to 1, with 0 representing the lowest similarity and 1 standing for the highest similarity. To each gene pair, we calculate five types of similarities based on five genomics data sources separately, obtaining (1) a network similarity derived from protein-protein interaction data, (2) a sequence similarity derived from protein sequence data, (3) an expression similarity derived from gene expression data, (4) a pathway similarity derived from gene annotations in the KEGG database [27], and (5) a semantic similarity derived from gene annotations in the biological process domain of the Gene Ontology [26].

#### Network similarity

We retrieved a total of 34,364 manually curated PPIs among 8,919 proteins from the HPRD database [32]. Treating proteins as nodes and interactions between proteins as edges, we obtain a sparse protein-protein interaction network. For each pair of two proteins *g* and *g’*, we calculate the length of the shortest path (*L*_*gg’*_) between the proteins and define the network similarity between the proteins using a Gaussian kernel, as

Sgg'PPI=exp−Lgg'2.

Mapping proteins back to genes, we obtain network similarities between a total of 8,919 genes.

#### Sequence similarity

We downloaded sequences of the 8,919 genes in the HPRD database from the NCBI Refseq database. Applying the NCBI blastp program [33] with default settings, we obtain an e-value (*e*_*gg’*_) for each pair of genes *g* and *g’*. All calculated e-values are then transformed and normalized to obtain sequence similarities between the genes, as

Sgg'SEQ=−logegg'/max−logegg'g,g'egg'>01egg'=0.

#### Expression similarity

Using whole-genome microarrays that targeting 44,775 human transcripts, an extensive gene expression atlas of 79 human tissues has been derived by Su *et.al*[34] and served as one of the largest quantitative evaluations of gene expression focusing on the protein-encoding transcriptome. With this data source, we calculate expression values of a gene (*g*) in the tissues and further constructed a 79-dimensional vector (*e*_*g*_) that represents expression levels of the gene across the tissues. Then, for any two genes *g* and *g’* in the 8,919 genes, we calculate the absolute Pearson correlation coefficient of the corresponding vectors (*e*_*g*_ and *e*_*g’*_) to obtain the expression similarity $S_{gg^{\prime }}^{\mathrm{EXP}}$ between the genes, as

$$S_{gg^{\prime }}^{\mathrm{EXP}}=\left|\frac{\mathrm{cov}\left(e_{g},e_{g'}\right)}{\sigma \left(e_{g}\right)\sigma \left(e_{g'}\right)}\right|.$$

#### Pathway similarity

We downloaded annotations of all human pathways from the KEGG database (released in July 2011). After removing all diseases-related pathways to avoid bias towards well-studied diseases, we obtain a total of 177 pathways. In the selected 8,919 genes, there are a total of 2,604 genes included in these pathways. For each of these genes (*g*), we construct a 177-dimensional binary vector (*P*_*g*_), with an element being 1 if the gene is involved in the corresponding pathway and 0 otherwise. Then, for a pair of two genes *g* and *g’*, we calculate the cosine of the angle between the corresponding vectors (*P*_*g*_ and P_*g*’_) to obtain the pathway similarity between the two genes, as

Sgg'KEGG=pg⋅pg'PgPg',

where |*P*_*g*_| and |*P*_*g* '_| are the norms of the vectors *P*_*g*_ and *P*_*g’*_, respectively.

#### Semantic similarity

We downloaded the biological process domain of the gene ontology (GO) and associated annotations from the gene ontology project [26]. Among the 8,919 genes, 5,549 genes have at least one biological process annotation. With this data source, we calculate the semantic similarity between any pair of the 5,549 genes using the tool GOSemSim [35,36]. Briefly, for a term *t* in the biological process domain, the probability that the term is used in annotations is estimated as

$$p_{t}=\frac{\#\left\{\mathrm{term}\;t\;\mathrm{or}\;\mathrm{its}\;\mathrm{descendants}\;\mathrm{used}\;\mathrm{in}\;\mathrm{annotations}\right\}}{\#\left\{\mathrm{Total}\;\mathrm{annotations}\right\}}.$$

Then, the semantic similarity of two terms *t* and *t’* is defined as the information content of their lowest common ancestor, as

$$S_{tt^{\prime }}=-\log\min_{x\in A\left(t,t^{\prime }\right)}p_{x},$$

where *A*(*t,t’*) is the set of all common ancestors of *t* and *t’*. Then, for two genes *g* and *g’*, represented as two sets of GO terms *G* and *G’* , respectively, their semantic similarity is calculated as

$$S_{gg^{\prime }}^{\mathrm{GOBP}}=\frac{1}{\left|G\right|+\left|G^{\prime }\right|}\left(\sum_{t\in G}\max_{t^{\prime }\in G^{\prime }}S_{tt^{\prime }}+\sum_{t^{\prime }\in G}\max_{t\in G}S_{tt^{\prime }}\right).$$

#### Dealing with missing data

As mentioned above, values in gene similarity range from 0 to 1, where 0 for the lowest similarity and 1 for the highest similarity. However, some similarity scores between genes could not be calculated, as shown above in KEGG pathways and GO annotations, yielding the missing data problem. To deal with this problem, we simply replaced missing values with the lowest similarity value 0.

#### Phenotypic similarity

We obtained phenotypic similarities between 5,080 human diseases from the literature [31]. Briefly, van Driel *et al*. used the anatomy (A) and the disease (C) sections of the medical subject headings vocabulary (MeSH) to extract terms from the OMIM database, and characterized each disease phenotype with a vector of standardized and weighted phenotypic feature terms mapped from corresponding OMIM records in the full text (TX) and clinical synopsis (CS) fields. Then, they calculated for each pair of disease phenotypes a similarity score as the cosine of the angle between feature vectors corresponding to the diseases. They further evaluated the reliability of the resulting phenotypic similarities and showed that disease similarity scores are positively correlated with a number of measures of gene functions.

### Linear regression with lasso penalty

We adopt a multiple linear regression model to explain the phenotypic similarity between two diseases *d* and *d’* using genes associated with the diseases, as $S_{dd^{\prime }}=\alpha_{d}+\sum_{i=1}^{5}\beta_{d}^{i}\left(\sum_{g\in G\left(d\right)}\sum_{g^{\prime }\in G\left(d^{\prime }\right)}S_{gg^{\prime }}^{i}\right)$_,_ where *G*(*d*) and *G*(*d’*) are sets of genes associated with *d* and *d’* , respectively. Parameters in this regression model can be estimated using the maximum likelihood estimation, which is equivalent to solving the following optimization problem by the least squares approach,

$$\min:{\sum_{\forall d^{\prime }\in D}\left[\left(\alpha_{d}+\sum_{i=1}^{5}\beta_{d}^{i}\left(\sum_{g\in G\left(d\right)}\sum_{g^{\prime }\in G\left(d^{\prime }\right)}S_{gg^{\prime }}^{i}\right)\right)\right]}^{2}.$$

To filter out unimportant data sources, a regularization term is added in the above objective function, as

$$\begin{array}{ll} \min: & {\sum_{\forall d^{\prime }\in D}\left[S_{dd^{\prime }}-\left(\alpha_{d}+\sum_{i=1}^{5}\beta_{d}^{i}\left(\sum_{g\in G\left(d\right)}\sum_{g^{\prime }\in G\left(d^{\prime }\right)}S_{gg^{\prime }}^{i}\right)\right)\right]}^{2}\\ & \;+\lambda \sum_{g\in G\left(d\right)}\sum_{i=1}^{5}R\left(\beta_{d}^{i}\right), \end{array}$$

where *λ* is a constant, and $R\left(\beta_{d}^{i}\right)$ serves as the regularization term. In this work, the lasso penalty $R\left(\beta_{d}^{i}\right)=\left|\beta_{d}^{i}\right|$ is adopted, with the benefit of shrinking some coefficients to zero and therefore serving as a feature selection method [37,38]. This property enables our method to retain good data sources as effective features in the calculation of scores for candidate genes. The lasso regression is calculated using a modified version of the LARS algorithm [37,38]. In each run, the value of *λ* is optimized as the one with best goodness-of-fit estimates AIC (Akaike's Information Criterion). The Matlab code of solving lasso regression is downloaded from the website http://www.di.ens.fr/~mschmidt/Software/lasso.html.

Given a query disease and a set of candidate genes, we then calculate a score for an individual candidate gene based on the above multiple regression model. Let *d* indicates the query disease and *g* a candidate gene, we assume the candidate gene is the only one associated with the disease, i.e. *G*(*d*) = {*g*}, and we rewrite the regression function as $S_{dd^{\prime }}=\alpha_{d}+\sum_{i=1}^{5}\beta_{d}^{i}\left(\sum_{g^{\prime }\in G\left(d^{\prime }\right)}S_{gg^{\prime }}^{i}\right)$_,_ where *d’* is any disease included in the phenotypic similarities. We then fit this model using the lasso penalty strategy and calculate the coefficient of determination (*R*^2^) of the fitted model as a score to measure the strength of association between the query disease (*d*) and the candidate gene (*g*). Repeating this procedure to obtain a score for each candidate gene, we are able to rank the candidate genes in non-increasing order according to their scores to obtain a ranking list.

### Validation methods and evaluation criteria

We adopt three large-scale leave-one-out cross-validation experiments to assess the capability of the proposed method in discovering disease genes. Using the tool BioMart [39], we obtain a total of 1,428 associations between 1,126 human diseases and 938 genes. In each validation run, we use a known disease-gene association as the test case, pretend that the association is unknown, and rank the test gene in the case against a set of control genes that are obtained in the following three different ways: 1) an artificial linkage interval (all neighboring genes within 10 Mb on both sides of the test gene are selected as control genes); 2) random controls (99 genes randomly selected from all the 8,919 genes are used as control genes); 3) the whole genome (all the 8,919 genes are used as control genes).

With ranks of test genes collected, we use the following criteria to evaluate the performance of the proposed method. First, we claim a prediction as success if a test gene is ranked first (having the largest *R*^2^ value), and we calculate the proportion of test genes that are successfully predicted. To eliminate the possible influence of the number of control genes, we calculate a criterion called *fold enrichment* (FE) [13,14]. If a method ranks a proportion of *p* known disease genes at the top of a total of *m* candidate genes, the fold enrichment is calculated as *pm*. For example, when testing against random controls, our method successfully ranks known disease genes at the top of a total of 100 candidate genes in 57.42% (820 of 1,428) test cases, achieving a fold enrichment of 57.42% × 100 = 57.42 (*p* = 57.42%, *m* = 100). Second, we normalize ranks by dividing them with the total number of candidate genes in the ranking list to obtain rank ratios and calculate a criterion called mean rank ratio (MRR) as the average of rank ratios of all test genes in the validation runs. Third, given a threshold of rank ratio, we calculate the criterion of *sensitivity* as the fraction of test genes ranked above the threshold and the criterion of *specificity* as the fraction of control genes ranked below the threshold, and we further plot the rank receiver operating characteristic (ROC) curve by varying threshold values and calculated the area under the ROC curve (AUC). Finally, given a threshold value of the *R*^2^ value, we considered candidate genes whose *R*^2^ value were greater than the threshold as positive predictions and obtained a set of true positives as the intersection of test genes and the positive predictions. We then define the criterion of *recall* as the fraction of the true positives in the test genes and the criterion of *precision* as the fraction of the true positives in all positive predictions.

It may be a concern that a disease is associated with multiple genes. Intuitively, the inclusion of known relationships between a disease and its associated genes may facilitate the identification of novel genes that are associated with the disease. To eliminate such a confounding factor, we perform *ab initio* predictions to assess the capability of our method in discovering genes that are associated with a disease whose genetic basis is completely unknown. Specifically, in each prediction run, we use a known disease-gene association as the test case, suppose that all associations of the test disease involved in are unknown, and rank the test gene in the case against a set of control genes. Similar to the leave-one-out cross-validation experiments, we also use three control sets: an artificial linkage interval, random controls, and the whole genome. We consider a prediction as success if the test gene is ranked among top *k* of the ranking list, and calculate the criteria as defined previously. When *k* is equal to 1 (the test disease is associated with a single gene), an *ab initio* prediction degenerates to a leave-one-out cross-validation.

## Results

### Performance of BRIDGE

**Figure 2:**
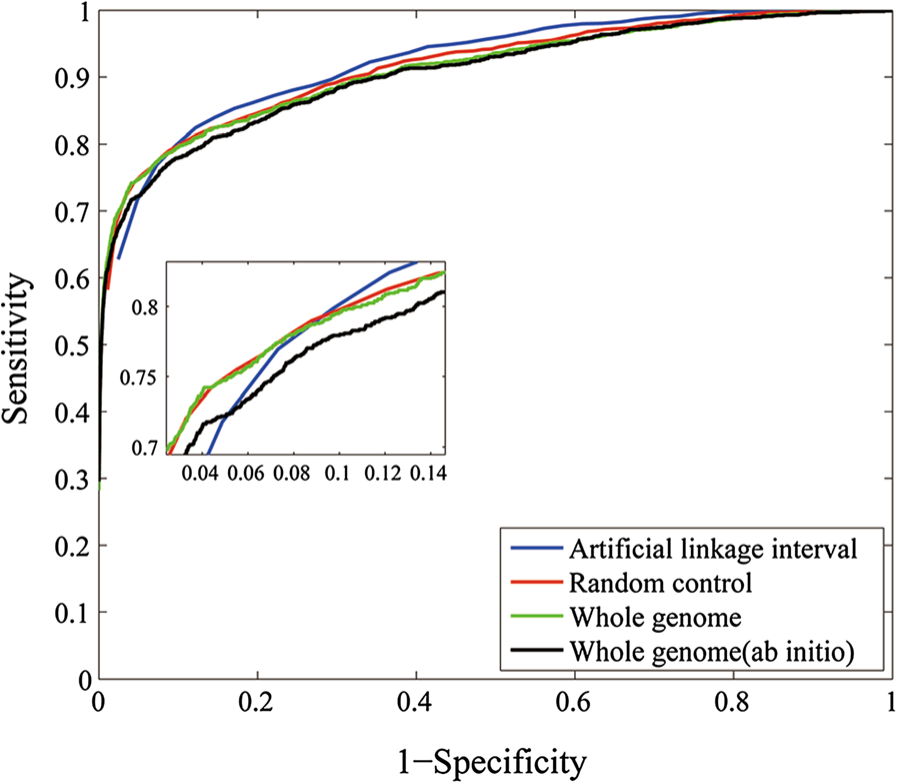
**The leave-one-out cross-validation results.** ROC curve on artificial linkage interval, random control, whole genome are shown. The *ab initio* prediction result on whole genome is also shown as black line. The zoom-in plot shows details of the low 1-specificity region.

**Figure 3:**
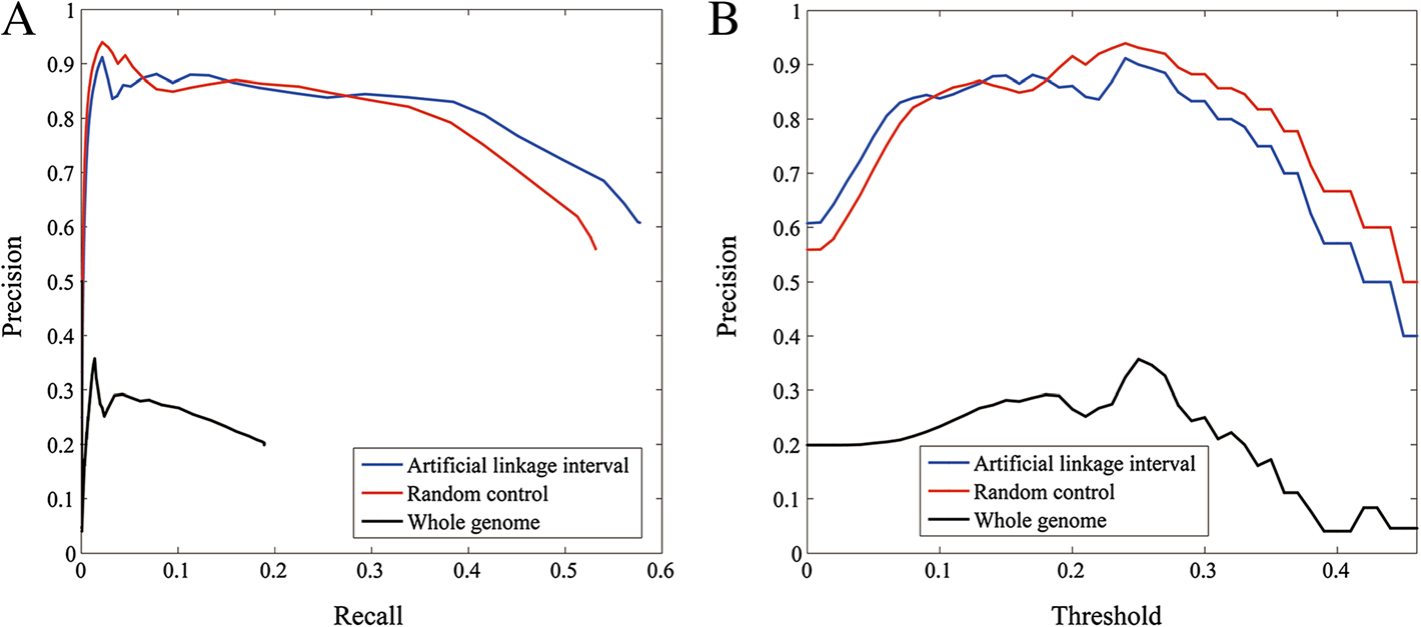
**The performance of BRIDGE on three control sets. (A)** The precision- recall curve when score threshold varies. **(B)** Score threshold plotted against precision.

We first carried out the leave-one-out cross-validation experiment against artificial linkage intervals to simulate the capability of BRIDGE in discovering disease-associated genes from a candidate region identified by traditional disease-mapping methods such as linkage analysis and association studies [40,41]. Focusing on the 1,428 associations between 1,126 diseases and 938 genes, in each validation run, we selected a test case of disease-gene associations out of the 1,428 known disease-gene associations and collected a set of control genes that were located within 10 Mbp around the gene in the case (i.e., test gene). We found that the number of control genes varied from 15 to 58 in these artificial linkage intervals, with the median being 39. We then ranked each test gene against its control gene set. Results show that 892 out of the 1,428 (62.47%) test genes are ranked at top one. Moreover, the mean rank ratio (MRR) of all 1,428 test genes is only 8.90%, suggesting that most test genes are assigned high ranks. We further varied the rank threshold, calculated the sensitivity and specificity under each threshold value, and plotted the ROC curve in Figure 2. It can be clearly seen from the figure that the curve (blue line) increases rapidly towards the top-left corner of the plot, indicating the effectiveness of our method in discovering disease-associated genes in this simulation study. The area under the ROC curve (AUC score) is calculated as 90.86%, also suggesting the validity of our method. We further took the goodness-of-fit of the linear regression model into consideration. By setting threshold values for the coefficient of determination (*R*^2^), we calculated the precision and the recall at each cutoff value, and we plotted the precision values under various threshold in Figure 3A. It can be seen from the figure that the curve exhibits a unimodal pattern, with the highest precision achieved as 91.18% at the *R*^2^ cutoff value of 0.02. We further plotted the precision-recall curve in Figure 3B, which demonstrates that the high precision can be maintained in a wide range of the recall. For example, the precision is as high as 84.7% when the recall maintains a relatively high value of 45%.

To assess the capability of BRIDGE in discovering disease-associated genes that spread over the genome, we conducted the leave-one-out cross-validation experiment against random controls by prioritizing each test gene against 99 genes selected at random. Results show that 820 out of the 1,428 (57.42%) test genes are ranked at the top. Moreover, the MRR of all 1,428 test genes is only 8.34%, suggesting that most test genes are also assigned high ranks. We further plotted the ROC curve in Figure 2 and calculated the AUC score as 90.78%, also suggesting the high quality of our method. The trend of the precision against the threshold *R*^2^ value and the precision-recall curve, as shown in Figure 3, also demonstrate similar patterns as those for the validation against an artificial linkage interval, revealing the effectiveness of our method in discovering disease-associated genes spreading over the genome.

We further pursued a more ambitious goal of scanning the whole genome for disease-associated genes by prioritizing each test gene against all 8,919 genes in the similarity profiles. Results show that 419 out of the 1,428 (29.34%) test genes are ranked at top one. We further plotted the ROC curve in Figure 2 and calculated the AUC score as 90.21%, also suggesting the high quality of our method. In order to simulate the situation of discovering disease-associated genes for diseases whose genetic basis is completed unknown, we further conducted the *ab initio* prediction experiments. Results show that 398 out of the 1,428 (27.87%) test genes are ranked first. Moreover, the AUC score only slightly drops to 89.16%, suggesting that BRIDGE does not rely on known genetic basis of a disease for future discovery and is effective in predicting genes for diseases without any known genetic origins.

### Contributions of individual data sources to gene prioritization

We further assessed the capability of BRIDGE in the prioritization of candidate genes using individual data sources and presented the results in Table 1. It can be seen from the table that each data source individually characterizes functional similarities between genes from a certain perspective, and thus shows positive contribution in the prioritization of candidate genes. For example, using the PPI data alone, BRIDGE achieves an AUC of 86.08% in the validation of linkage intervals and 82.23% in the validation of random controls. Using gene expression data alone, BRIDGE achieves an AUC of 74.30% in the validation of linkage intervals and 69.04% in the validation of random controls. Using other data sources, BRIDGE achieves AUC scores between 76.02% and 79.08% in the validation of linkage intervals. These results suggest that the PPI data provide more useful information in disease gene prioritization. This conjecture is also supported by the other criteria. For example, in the validation of linkage intervals, 47.41% test genes are ranked first by using PPI data alone, while only 23.25% are ranked first by using gene expression data (see Table 1 for more details).

**Table 1:** Validation results of each dataset and integration of 5 data sources

		**Artificial linkage interval**			**Random control**					**Whole genome**			
		**TOP**	**AUC**	**FE**	**MRR**	**TOP**	**AUC**	**FE**	**MRR**	**TOP**	**AUC**	**FE**	**MRR**
ALL (Lasso)	Leave one out	62.47%	90.86%	68.09	8.90%	57.42%	90.78%	57.42	8.34%	29.34%	90.21%	2616.84	7.57%
	*Ab initio*	61.48%	90.61%	67.01	10.98%	56.58%	90.49%	56.58	8.87%	27.87%	89.16%	2484.89	8.09%
ALL (Regular)	Leave one out	56.96%	89.92%	64.25	11.20%	53.69%	90.24%	53.69	9.98%	24.16%	89.01%	2154.80	8.72%
	*Ab initio*	55.47%	89.52%	63.74	11.60%	52.64%	90.02%	52.64	9.65%	23.11%	88.16%	2061.20	9.22%
PPI	Leave one out	47.41%	86.08%	51.66	16.94%	42.07%	82.23%	42.07	18.05%	9.87%	79.78%	880.31	19.46%
	*Ab initio*	44.82%	84.45%	48.85	17.95%	39.29%	80.02%	39.29	20.29%	9.16%	79.05%	816.98	20.08%
KEGG	Leave one out	42.16%	76.02%	46.20	16.36%	35.89%	73.12%	35.89	19.50%	11.48%	62.38%	1023.90	12.93%
	*Ab initio*	41.02%	75.19%	44.91	16.52%	34.20%	71.31%	34.20	20.10%	11.20%	62.20%	998.93	13.01%
GS	Leave one out	40.34%	79.08%	43.97	18.59%	40.20%	74.07%	40.20	15.80%	10.64%	67.26%	948.98	15.09%
	*Ab initio*	40.27%	78.69%	43.89	18.80%	38.45%	72.73%	38.45	16.40%	10.31%	66.03%	919.55	15.65%
GE	Leave one out	23.25%	74.30%	25.34	31.15%	17.44%	69.04%	17.44	29.51%	1.75%	67.61%	156.08	28.79%
	*Ab initio*	22.97%	74.03%	25.04	31.48%	16.53%	68.60%	16.53	29.94%	1.47%	67.12%	131.11	29.21%
GO	Leave one out	24.65%	76.99%	26.86	24.94%	20.24%	69.18%	22.24	23.51%	8.05%	61.74%	717.98	22.63%
	*Ab initio*	24.00%	76.81%	26.16	25.08%	20.10%	68.89%	20.13	23.71%	7.47%	61.44%	666.25	22.95%

We also noticed the great improvement in the performance of BRIDGE when the five data sources were integrated. When applying a regular regression to integrate all of the five data sources, we achieved a TOP of 56.96%, an AUC of 89.92%, an FE of 64.25, and an MRR of 11.2% in the validation against linkage intervals. In the validation against random controls, the regular regression model achieved a TOP of 53.69%, an AUC of 90.24%, an FE of 53.69, and an MRR of 9.98%. In comparison, BRIDGE achieved a TOP of 62.47%, an AUC of 90.86%, an FE of 68.09, and an MRR of 8.90% in the validation against linkage, and a TOP of 57.42%, an AUC of 90.78%, an FE of 57.42, and an MRR of 8.34% in the validation against random controls. These results suggest that the lasso penalty is helpful for variable selection in the integration of multiple data sources, and thus benefiting our method to achieve high performance in pinpointing disease genes.

### Comparison with existing methods

We systematically compared BRIDGE with two existing methods CIPHER [13] and ENDEAVOUR [11]. Briefly, both BRIDGE and ENDEAVOUR were based on the integration of multiple genomic data, while ENDEAVOUR used more than ten data sources, including the five datasets (PPI, GS, GE, KEGG, and GO) used in our study. CIPHER was in principle similar to the regular regression method using only gene similarities derived from PPI data as the predictor variable. The comparisons were conducted by repeating the cross-validation experiments on both artificial linkage intervals and random control gene sets for each method, based on its preferred genomic datasets. The performance of each was evaluated using the four criteria (TOP, AUC, FE and MRR).

As shown in Table 2, in the validation against artificial linkage intervals, CIPHER achieved a TOP of 47.41%, an AUC of 86.08%, an FE of 51.66, and an MRR of 16.94%, while BRIDGE achieved a TOP of 62.47%, an AUC of 90.86%, an FE of 68.09, and an MRR of 8.90%. The improvement of BRIDGE over CIPHER in this validation experiment was then achieved as 15.06% for TOP, 4.78% for AUC, 16.43 for FE, and 8.04% for MRR. Similarly, in the validation against random controls, CIPHER achieved a TOP of 42.07%, an AUC of 82.23%, an FE of 42.07, and an MRR of 18.05%, while BRIDGE achieved a TOP of 57.42%, an AUC of 90.78%, an FE of 57.42, and an MRR of 8.34%. The improvement of BRIDGE over CIPHER is then achieved as 15.35% for TOP, 8.55% for AUC, 15.35 for FE, and 9.71% for MRR. These results clearly suggest that BRIDGE outperforms CIPHER in all criteria evaluated, indicating the power of integrating multiple data sources.

**Table 2:** Comparisons of BRIDGE with CIPHER and ENDEAVOUR

	**Artificial linkage interval**				**Random control**			
	**TOP**	**AUC**	**FE**	**MRR**	**TOP**	**AUC**	**FE**	**MRR**
CIPHER^1^	47.41%	86.08%	51.66	16.94%	42.07%	82.23%	42.07	18.05%
BRIDGE^1^	62.47%	90.86%	68.09	8.90%	57.42%	90.78%	57.42	8.34%
ENDEAVOUR^2^	45.80%	91.89%	49.92	7.79%	44.10%	92.03%	44.10	11.27%
BRIDGE^2^	66.15%	94.17%	72.10	6.73%	63.50%	93.85%	63.50	6.95%

^1^CIPHER and BRIDGE are evaluated using the 1,428 associations between 1,126 diseases and 938 genes. ^2^ENDEAVOUR and BRIDGE are evaluated using the 470 associations between 168 diseases and 375 genes.

ENDEAVOUR was developed according to the guilt-by-association principle, and thus a set of seed genes known to be associated with a query disease was required in the calculation of scores for candidate genes. To meet this requirement, we extracted from our data set a total of 470 associations between 168 diseases and 375 genes, with each of these diseases associated with 2 or more genes. We then compared ENDEAVOUR (online service at http://homes.esat.kuleuven.be/~bioiuser/endeavour) and BRIDGE on this data set. Briefly, in the leave-one-out cross-validation experiment against linkage intervals, ENDEAVOUR achieved a TOP of 45.80%, an AUC of 91.89%, an FE of 49.92, and an MRR of 7.79%. BRIDGE achieved a TOP of 66.15%, an AUC of 94.17%, an FE of 72.10, and an MRR of 6.73%. The improvement of BRIDGE over ENDEAVOUR in this validation experiment is then achieved as 20.35% for TOP, 2.28% for AUC, 22.18 for FE, and 1.06% for MRR. In the validation against random controls, ENDEAVOUR achieved a TOP of 44.10%, an AUC of 92.03%, an FE of 44.10, and an MRR of 11.27%, while BRIDGE achieved a TOP of 63.50%, an AUC of 93.85%, an FE of 63.50 and an MRR of 6.95%. The improvement of BRIDGE over ENDEAVOUR in this validation experiment is then achieved as 19.40% for TOP, 1.82% for AUC, 19.40 for FE, and 4.32% for MRR. These results clearly suggest that BRIDGE outperforms ENDEAVOUR in all criteria evaluated, though ENDEAVOUR used more data sources than BRIDGE.

### Case studies: obesity and type II diabetes mellitus

We further performed two case studies on obesity and diabetes mellitus to demonstrate the capability of BRIDGE in uncovering disease genes and predicting novel susceptible candidates. For each of these two disorders, we performed the *ab initio* whole-genome prediction and checked the predicted genes ranked in top 100. We chose 100 because this number was comparable to the resolution of a typical association study for human complex diseases [41,42].

Obesity is a major public health problem, resulting in increased morbidity and mortality and severe economic burdens on healthcare systems. Although several genes involved in obesity were reported to act through the central nervous system (CNS) [43], and in particular the hypothalamus, to influence energy balance and appetite, research on obesity is far from complete [44-46]. The overview section of obesity in OMIM (MIM: 601665) presented a list of 15 genes, 13 of which were characterized in our gene similarity profiles. We first examined the results of a genome-wide *ab-initio*-prioritization. BRIDGE assigned high ranks to most of the known obesity genes, with 8 out of these 13 in top 100 (actually in top 50) of the ranked genome. This was statistically significant compared to a uniform distribution of disease gene ranks (3.35-16, Fisher’s exact test, one-sided). Furthermore, we checked whether our method could predict novel susceptibility genes that have been identified only recently. We found 63 genes (Additional file 1: Table S1) suggested as disease genes and 28 genes reported to cause obesity or fatty liver annotated by DAVID [47] and GeneCards [48]. Taking genes LEP and NPY as examples, gene LEP was ranked 4th and gene NPY 6th in our prediction. We found numerous literature reports suggest that LEP (leptin) is a protein produced by adipose tissue that circulates to the brain and interacts with receptors in the hypothalamus to inhibit eating [49,50]. It has also been reported that stress exaggerates diet-induced obesity through a peripheral mechanism in the abdominal white adipose tissue that is mediated by NPY (neuropeptide Y) [51]. Actually, synthesis and release of NPY are both regulated by leptin binding to its hypothalamic receptor which mediates some of the effects of leptin on food intake [51,52]. Another susceptible gene is NPY2R, a 5′ variant of which has been reported to be associated with both severe adult obesity and childhood obesity by case–control studies [52,53]. Further, we examined gene function enrichment among the top 100 obesity-related genes by using DAVID to analyze enrichment of GO biological process terms. Results (Additional file 2: Table S2) show that those genes are enriched in fatty acid metabolic processes, lipid metabolic processes, and cell communication. These findings are consistent with the current knowledge on obesity [41,43].

Type II diabetes mellitus is a disease characterized by high blood glucose in the context of insulin resistance and relative insulin deficiency. The OMIM overview section of type II diabetes mellitus (MIM: 125853) presented a list of 29 genes, 20 of which are characterized in our gene similarity profiles. We first examined the results of a genome-wide *ab-initio*-prioritization. BRIDGE assigned high ranks to most of the known diabetes mellitus causing genes, with 13 out of these 20 in the top 100 of the ranked genome. This was statistically significant compared to a uniform distribution of disease gene ranks (1.08e-16, Fisher’s exact test, one-sided). Next we checked whether our method can predict novel susceptibility genes that were identified recently. We found 52 genes (Additional file 3: Table S3) suggested as disease genes and 33 genes were reported to be genes associated with diabetes, noninsulin-dependent diabetes mellitus (NIDDM) or isulin sensitivity annotated by DAVID and GeneCards. For example, gene INS was reported to be involved in insulin resistance [54,55], insulin sensitivity [56] and NIDDM [57]. IAPP was reported to be involved insulinoma, insulin resistance [58,59]. Furthermore, we examined gene functions enriched among the top 100 genes, carried out using DAVID. An analysis enrichment of GO biological process terms (Additional file 4: Table S4) showed that those genes were enriched in the insulin receptor signaling pathway, carbohydrate transport, carbohydrate homeostasis, and the carbohydrate metabolic process [55,60].

We also checked the genes RNF128 and MAFA, which were ranked at top 1 and top 2 for diabetes, respectively. RNF128 encodes a type I transmembrane protein located in the endocytic pathway and its expression significantly inhibited activation-induced IL4 and IL2 [61] that are involved in type I diabetes mellitus pathway (KEGG: hsa04940). MAFA is a transcription factor binding to RIPE3b, a conserved enhancer element that regulates pancreatic beta cell-specific expression of the insulin gene (INS) [62]. The above evidence not only supports the fact that MAFA and RNF128 are genes associated with diabetes, but also proposes more links and nodes to complete the maturity onset diabetes pathway (KEGG: hsa04950) and type I diabetes mellitus (KEGG: hsa04940).

Early studies have recognized that obesity and diabetes are related. Obesity confers considerable risk for diabetes and is found in approximately 55% of patients diagnosed with type II diabetes [63]. Central obesity is known to predispose individuals to insulin resistance [64,65]. Abdominal fat is especially active hormonally, secreting a group of hormones called adipokines that may impair glucose tolerance [64]. Our results further confirm that obesity and diabetes mellitus are partly related through associated genes and cellular processes [66]. Fifteen genes ranked among top 100 genes for obesity are also related to insulin sensitivity, NIDDM, insulin resistance. Meanwhile, 9 genes ranked among top 100 genes for diabetes are also related to obesity or fatty liver (annotated by DAVID database, Additional file 1: Table S1 and Additional file 3: Table S3). These genes are mostly involved in the regulation of energy balance, as annotated by DAVID database. These findings are consistent with early research suggesting that obesity and diabetes are highly related. The results also support that molecular therapy of obesity and diabetes may be done by controlling the genes involved in the regulation of energy balance [41,44].

### Predicted transcriptional networks involved in obesity and diabetes

Using our predicted results, we further analyzed transcription factors (TF) and dissected possible transcriptional networks involved in complex diseases. In the SABiosciences’ proprietary database known as DECODE (DECipherment Of DNA Elements) (http://www.sabiosciences.com/chipqpcrsearch.php), predicted binding sites of different transcription factors can be searched for promoter regions of human genes. We analyzed TFs of the top 100 predicted genes for obesity and diabetes. For obesity, 192 TFs (Additional file 1: Table S1) were predicted, with an average of 7 TFs per gene. We then linked all these TFs and their regulated genes to construct a predictive transcriptional network with a total of 292 nodes and 705 edges (Additional file 5: Figure S1). Among these TFs, we found 9 TFs (PPAR-gamma1, PPAR-gamma2, NF-kappaB, NF-kappaB1, GR, GR-beta, GR-alpha, c-Jun, AP-1) regulating more than 21 genes individually.

For diabetes, 182 TFs (Additional file 3: Table S3) were predicted, with an average of 6.6 TFs per gene. We also linked these TFs and regulated genes to construct a predictive transcriptional network with a total of 282 nodes and 664 edges (Additional file 6: Figure S2). Among these TFs, we found 9 TFs (PPAR-gamma1, PPAR-gamma2, NF-kappaB, NF-kappaB1, HNF-4alpha1, NRSF form1, NRSF form2, c-Jun, AP-1) regulating more than 12 genes individually. Most of these common TFs (PPAR-gamma1, PPAR-gamma2, NF-kappaB, NF-kappaB1, c-Jun, AP-1) are related to metabolic and neurological processes, further confirming that a similar underlying regulatory mechanism exists for both obesity and diabetes.

Beside these enriched transcription factors, other oncogenic transcription factors, such as P53 and E2F that are involved in many types of cancers, are also found to regulate the predicted genes for obesity and diabetes. We observed that many of the top 100 predicted genes of obesity were associated with many kinds of diseases. Our study was consistent with recent findings, suggesting that obesity and cancer may share a common fatty acid network [67] and obesity may cause many other diseases, such as diabetes, leukemia, colon cancer and breast cancer.

## Discussion and conclusion

In this paper, we developed a method called BRIDGE to integrate the disease phenotypic similarity with such functional genomic data sources as protein-protein interactions, gene sequence similarity, gene expression profiles, gene pathway annotations, and gene ontology annotations to prioritize candidate genes for the discovery of disease genes. We proposed to convert each genomic data source into a pairwise similarity profile describing functional similarity of genes and then use a multiple regression model to characterize the strength of association between a candidate gene and a query disease. A lasso penalty was further incorporated to achieve automatic selection of the most valuable information in the regression process. Through large-scale validation experiments, we demonstrated that the proposed method was capable of ranking a large proportion of known disease-associated genes in the top of ranking lists, suggesting the superior performance of our method. We further performed detailed case studies on obesity and diabetes to illustrate potential novel findings produced by our method.

The success of our method is mainly due to the integrated use of multiple genomics data. As we have shown, each data source characterizes a certain aspect of functional similarity between genes, and through the integration framework, multiple data sources are able to complement each other to achieve a more comprehensive description of functional similarity between genes. Another merit characteristic of our method is the capability of predicting genes associated with diseases whose genetic bases are completely unknown. This feature is achieved by using the phonotypic similarities. Although there have been a few methods using such this information, BRIDGE demonstrates superior performance in prediction tasks, mainly owing to the use of the lasso regression model that enables the automatic selection of valuable information.

The following aspects of our method may be further improved. First, the computational burden of the lasso regression method is obviously heavier than the ordinary regression method. Second, a problem in the study of associations between diseases and genes is the underlying bias towards well-studied genes [68]. Existing methods for candidate gene prioritization still do not have a way to correct such bias. With the integration of multiple omics data sources, however, this bias issue is alleviated, because different data sets measure gene functions from different perspectives, and thus final results will not depend on a single data source. Nevertheless, how to completely eliminate the influence of bias is still an open question worth further exploration. Finally, in this paper we demonstrated the integration of five data sources. However, it is not hard to incorporate more data sources into the BRIDGE framework. These data sources may include but not limited to literature information (abstracts in EntrezGene), SNP data, EST expression (EST data from Ensemble) [69], protein domains (Pfam and InterPro) [70], *cis*-regulatory modules (TOUCAN) [71], transcriptional motifs (TRANSFAC) [72], and many others. The main work in this possible extension will be the derivation of suitable pairwise similarities for genes from these data sources.

## Competing interests

The authors declare that they have no competing interests.

## Authors’ contributions

RJ provide guidance and planning for this project. YC produced programs, analyzed main results and wrote the manuscript. XW contributed in preparing some data and results analysis. All authors read and approved the final manuscript.

## Pre-publication history

The pre-publication history for this paper can be accessed here:

http://www.biomedcentral.com/1755-8794/6/57/prepub

## Supplementary Material

Additional file 1: Table S1 Detailed information of predicted top 100 obesity genes. Click here for file

Additional file 2: Table S2 Enriched biological processes in top 100 obesity related genes. Click here for file

Additional file 3: Table S3 Detailed information of predicted top 100 diabetes genes. Click here for file

Additional file 4: Table S4 Enriched biological processes in top 100 diabetes related genes. Click here for file

Additional file 5: Figure S1 The predicted transcription network of 100 predicted obesity genes. The network was constructed by top 100 predicted genes (red) for obesity and their related 192 transcription factors (pink). The transcription factors regulating more than 21 genes are noted as green. Click here for file

Additional file 6: Figure S2 The predicted transcription network of 100 predicted diabetes genes. The network was constructed by top 100 predicted genes (red) for diabetes and related 182 transcription factors (pink). The transcription factors regulating more than 12 genes are noted as green. Click here for file

## Web resources

The URLs for data presented herein are as follows:

BRIDGE web server, http://bioinfo.au.tsinghua.edu.cn/jianglab/bridge/

Online Medelian inheritance in Man (OMIM), http://www.ncbi.nlm.nih.gov/Omim

Reference Sequence (RefSeq), http://www.ncbi.nlm.nih.gov/RefSeq/

Gene Expression Omnibus (GEO), http://www.ncbi.nlm.nih.gov/geo/

Kyoto Encyclopedia of Genes and Genomes (KEGG), http://www.genome.jp/kegg/

Human Protein Reference Database (HPRD), http://www.hprd.org/
