# Supplementary figures and images for: Integrating human omics data to prioritize candidate genes
Source: BMC Med Genomics. 2013 Dec 18;6:57. doi: 10.1186/1755-8794-6-57 (PMC3878333; doi:10.1186/1755-8794-6-57)

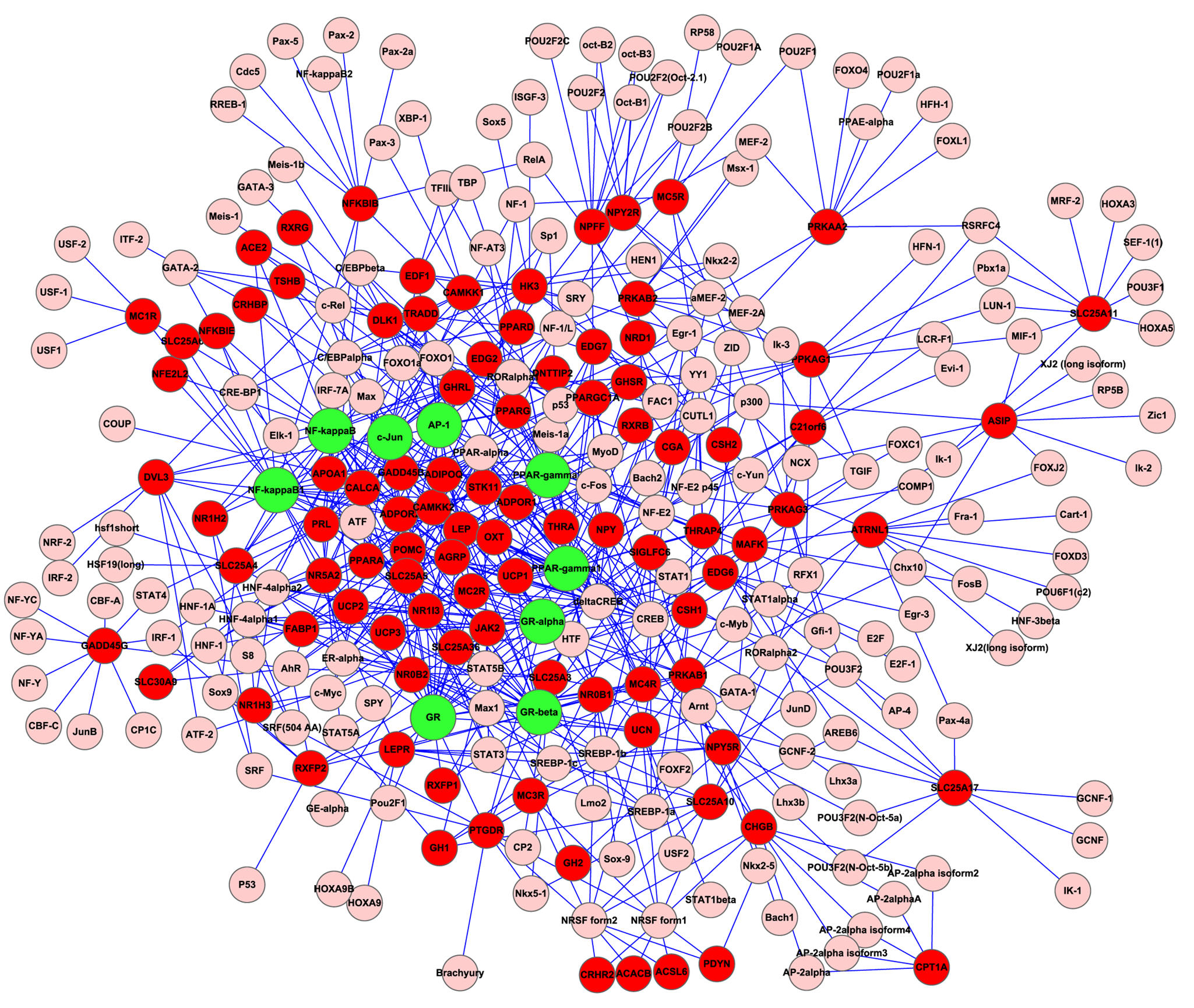

Supplement: Additional file 5: Figure S1 — The predicted transcription network of 100 predicted obesity genes. The network was constructed by top 100 predicted genes (red) for obesity and their related 192 transcription factors (pink). The transcription factors regulating more than 21 genes are noted as green. [file 1755-8794-6-57-S5.tiff]

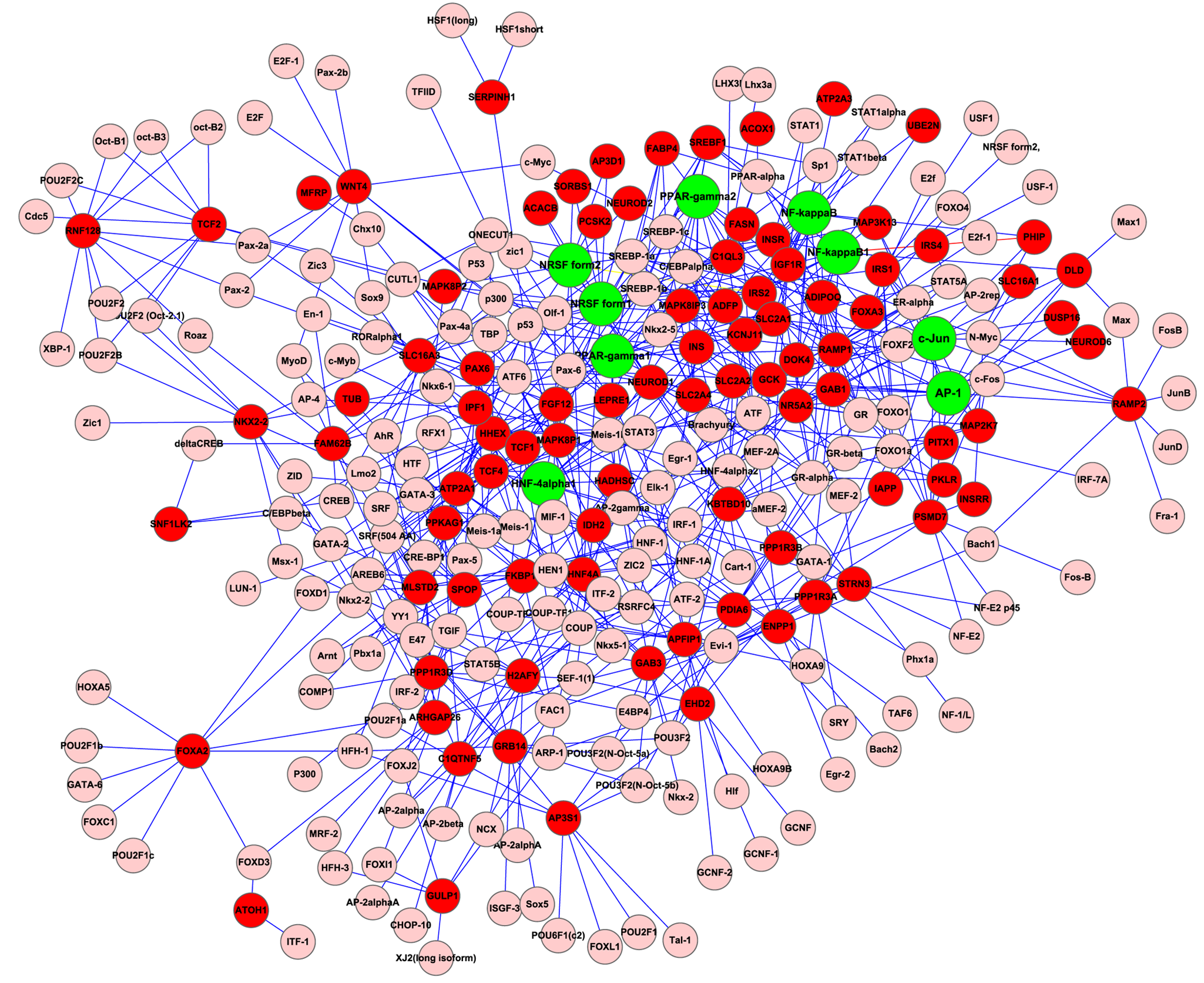

Supplement: Additional file 6: Figure S2 — The predicted transcription network of 100 predicted diabetes genes. The network was constructed by top 100 predicted genes (red) for diabetes and related 182 transcription factors (pink). The transcription factors regulating more than 12 genes are noted as green. [file 1755-8794-6-57-S6.tiff]
